# N-Terminal Pro-Brain Natriuretic Peptide Is Associated with a Future Diagnosis of Cancer in Patients with Coronary Artery Disease

**DOI:** 10.1371/journal.pone.0126741

**Published:** 2015-06-05

**Authors:** José Tuñón, Javier Higueras, Nieves Tarín, Carmen Cristóbal, Óscar Lorenzo, Luis Blanco-Colio, José Luis Martín-Ventura, Ana Huelmos, Joaquín Alonso, Álvaro Aceña, Ana Pello, Rocío Carda, Dolores Asensio, Ignacio Mahíllo-Fernández, Lorenzo López Bescós, Jesús Egido, Jerónimo Farré

**Affiliations:** 1 Department of Cardiology, IIS-Fundación Jiménez Díaz, Madrid, Spain; 2 Autónoma University, Madrid, Spain; 3 Laboratory of Vascular Pathology, IIS-Fundación Jiménez Díaz, Madrid, Spain; 4 Department of Cardiology, Hospital Clínico Universitario San Carlos, Madrid, Spain; 5 Department of Cardiology, Hospital Universitario de Móstoles, Madrid, Spain; 6 Department of Cardiology, Hospital de Fuenlabrada, Madrid, Spain; 7 Rey Juan Carlos University, Alcorcón, Madrid, Spain; 8 Department of Cardiology, Hospital Universitario Fundación Alcorcón, Madrid, Spain; 9 Department of Biochemistry, IIS-Fundación Jiménez Díaz, Madrid, Spain; 10 Department of Preventive Medicine, IIS-Fundación Jiménez Díaz, Madrid, Spain; 11 CIBERDEM, Madrid, Spain; Kaohsiung Chang Gung Memorial Hospital, TAIWAN

## Abstract

**Objective:**

Several papers have reported elevated plasma levels of natriuretic peptides in patients with a previous diagnosis of cancer. We have explored whether N-terminal pro-brain natriuretic peptide (NT-proBNP) plasma levels predict a future diagnosis of cancer in patients with coronary artery disease (CAD).

**Methods:**

We studied 699 patients with CAD free of cancer. At baseline, NT-proBNP, galectin-3, monocyte chemoattractant protein-1, soluble tumor necrosis factor-like weak inducer of apoptosis, high-sensitivity C-reactive protein, and high-sensitivity cardiac troponin I plasma levels were assessed. The primary outcome was new cancer diagnosis. The secondary outcome was cancer diagnosis, heart failure requiring hospitalization, or death.

**Results:**

After 2.15±0.98 years of follow-up, 24 patients developed cancer. They were older (68.5 [61.5, 75.8] vs 60.0 [52.0, 72.0] years; p=0.011), had higher NT-proBNP (302.0 [134.8, 919.8] vs 165.5 [87.4, 407.5] pg/ml; p=0.040) and high-sensitivity C-reactive protein (3.27 [1.33, 5.94] vs 1.92 [0.83, 4.00] mg/L; p=0.030), and lower triglyceride (92.5 [70.5, 132.8] vs 112.0 [82.0, 157.0] mg/dl; p=0.044) plasma levels than those without cancer. NT-proBNP (Hazard Ratio [HR]=1.030; 95% Confidence Interval [CI]=1.008-1.053; p=0.007) and triglyceride levels (HR=0.987; 95%CI=0.975-0.998; p=0.024) were independent predictors of a new cancer diagnosis (multivariate Cox regression analysis). When patients in whom the suspicion of cancer appeared in the first one-hundred days after blood extraction were excluded, NT-proBNP was the only predictor of cancer (HR=1.061; 95%CI=1.034-1.088; p<0.001). NT-proBNP was an independent predictor of cancer, heart failure, or death (HR=1.038; 95%CI=1.023-1.052; p<0.001) along with age, and use of insulin and acenocumarol.

**Conclusions:**

NT-proBNP is an independent predictor of malignancies in patients with CAD. New studies in large populations are needed to confirm these findings.

## Background

Patients with coronary artery disease (CAD) are not only at risk of developing cardiovascular events, but may also develop malignancies. Cancer shares some risk factors with CAD, as age, smoking, and even some dietary patterns could lead to the development of both disorders [1–3]. Therefore, finding biomarkers that predict risk of cancer in addition to that of cardiovascular events could be useful in CAD patients.

Natriuretic peptides are secreted by cancer cells [4,5] and N-terminal fragment of pro-brain natriuretic peptide (NT-proBNP) levels are increased in patients with cancer [6]. However, it has not been demonstrated whether NT-proBNP may predict the appearance of malignancies.

In order to study if increased NT-proBNP plasma levels predict cancer, we studied 704 patients with CAD who were free of malignancies at baseline. We also tested these biomarkers: monocyte chemoattractant protein-1 (MCP-1) and soluble tumor necrosis factor-like weak inducer of apoptosis (sTWEAK), both involved in inflammation and atherothrombosis, among other processes [7–9]; galectin-3, related to malignancies, heart failure, thrombosis, and renal dysfunction [10,11]; and high-sensitivity cardiac troponin I, which has been described to have prognostic value in stable CAD [12]. High-sensitivity C-reactive protein was studied as a reference given the large amount of information published on this biomarker.

## Methods

### Patients

The BACS & BAMI (Biomarkers in Acute Coronary Syndrome & Biomarkers in Acute Myocardial Infarction) studies included patients admitted to the Madrid-area hospitals Fundación Jiménez Díaz, Fuenlabrada, Móstoles, and Alcorcón with either non-ST elevation acute coronary syndrome or ST-elevation myocardial infarction. Inclusion criteria have been detailed previously [13]. Exclusion criteria were: age over 85 years, coexistence of other significant cardiac disorders except left ventricular hypertrophy secondary to hypertension, coexistence of any condition that could limit patient survival, impossibility to perform revascularization when indicated, patients who were not clinically stable at day six of admission, and subjects in whom follow-up was not possible.

### Ethics Statement

The research protocol conforms to the ethical guidelines of the 1975 Declaration of Helsinky as reflected in a priori approval by the human research committees of the institutions participating in this study: Fundación Jiménez Díaz, Hospital Fundación Alcorcón, Hospital de Fuenlabrada y Hospital Universitario de Móstoles. All patients signed informed consent documents.

### Study Design

In addition to plasma withdrawal at discharge, in the BACS & BAMI studies a second plasma sample was extracted 6–12 months later, on an outpatient basis. This paper is a sub-study of the BACS & BAMI studies, and reports the clinical and analytical findings obtained at this second plasma extraction, relating them to subsequent follow-up. Then, the moment of this second plasma extraction is considered to be the baseline visit of this sub-study. At this point, all patients were free of a diagnosis of cancer and even of any symptoms or signs suggestive of this diagnosis.

**Fig 1:**
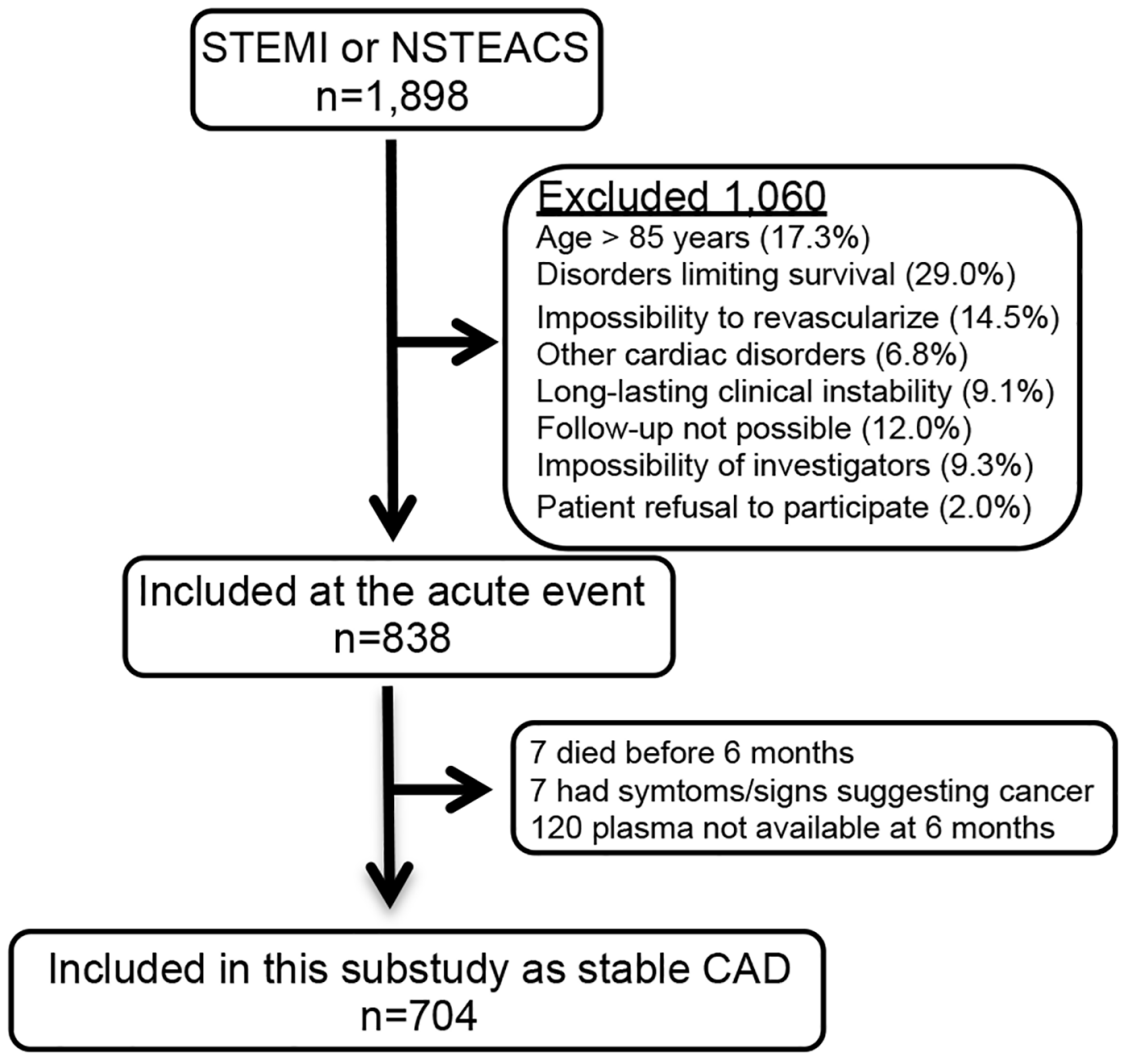
Recruitment flow-chart. **CAD:** Coronary artery disease. **NSTEACS:** Non-ST elevation Acute Coronary Syndrome. **STEMI:** ST-elevation myocardial infarction.

Between July 2006 and April 2010, 1,898 patients were discharged from the participant hospitals with a diagnosis of non-ST elevation acute coronary syndrome or ST-elevation acute myocardial infarction. Of these, 704 were eligible for this sub-study (Fig 1). The baseline visit and blood extraction for this sub-study took place between January 2007 and February 2011. The last follow-up visits took place in May 2012.

The primary outcome was the development of a new cancer with histological confirmation, excluding non-melanocytic skin tumors. The secondary outcome was the combination of new cancer, death, or heart failure requiring hospital admission.

At baseline, clinical variables were recorded and 12-hour fasting venous blood samples were withdrawn and collected in Ethylene-Diamine-Tetra-Acetic Acid. Blood samples were centrifuged at 2,500 g for 10 minutes and plasma was stored at -80°C. Patients were seen every year at their hospital. At the end of follow-up (maximum 4.6 years) the medical records were reviewed and patient status was confirmed by telephone contact.

### Analytical Studies

The investigators who performed the laboratory determinations (OL, LBC, JMV, and DA) were unaware of the clinical data. Plasma concentrations of MCP-1, galectin-3, and sTWEAK were determined in duplicate using commercially available enzyme-linked immunosorbent assay kits (BMS279/2 Bender MedSystems; DCP00, R&D Systems; BMS2006INST, Bender MedSystems; respectively) following the manufacters’ instructions. Intra- and inter-assay coefficients of variation were 4.6% and 5.9% for MCP-1, 6.2% and 8.3% for galectin-3, and 6.1% and 8.1% for sTWEAK, respectively. High-sensitivity C-reactive protein was assessed by latex-enhanced immunoturbidimetry (ADVIA 2400 Chemistry System, Siemens, Germany), NT-pro-BNP by immunoassay (VITROS, Orthoclinical Diagnostics, U.S.A.), and high-sensitivity cardiac troponin I was assessed by direct quimioluminiscence (ADVIA Centaur, Siemens, Germany). Lipids, glucose, and creatinin were determined by standard methods (ADVIA 2400 Chemistry System, Siemens, Germany).

### Statistical Analysis

Quantitative data did not follow a normal distribution and are displayed as median (interquartile range) and compared using the Mann-Whitney test for two independent samples and by the Kruskal-Wallis test for three or more samples. Qualitative variables are displayed as percentages and compared by χ^2^ or Fisher exact test when appropriate. Univariate Cox regression analysis was used to study the predictive power of the biomarkers studied along with these variables: age, sex, body-mass index, diabetes, smoking, hypertension, systolic blood pressure, previous heart failure, peripheral artery disease, cerebrovascular events, atrial fibrillation, ejection fraction<40%, LDL (low-density lipoprotein) cholesterol, HDL (high-density lipoprotein) cholesterol, triglycerides, non-HDL cholesterol, glycemia, glomerular filtration rate (Chronic Kidney Disease Epidemiology Collaboration method); therapy with aspirin, clopidogrel, acenocumarol, statins, oral antidiabetic drugs, insulin, angiotensin-converting enzyme inhibitors, angiotensin receptor blockers, aldosterone receptor blockers, betablockers, and diuretics; type of last acute coronary event, number of diseased vessels, use of percutaneous revascularization, drug-eluting stents, coronary artery by-pass graft, and existence of complete revascularization at that event.

Multivariate Cox regression analysis was performed with forward, stepwise selection, including the biomarkers studied and all the variables mentioned above, in order to assess which variables were independently associated with the primary and the secondary outcomes. Variables with p<0.05 were entered into the model, and those with p>0.10 were removed. Univariate and multivariate linear regression analyses were performed to assess the relationship of NT-proBNP plasma levels with age, glomerular filtration rate, triglycerides and the other biomarkers studied. Analyses were performed with SPSS 19.0 (SPSS Inc., New York).

## Results

### NT-proBNP and cancer

**Fig 2:**
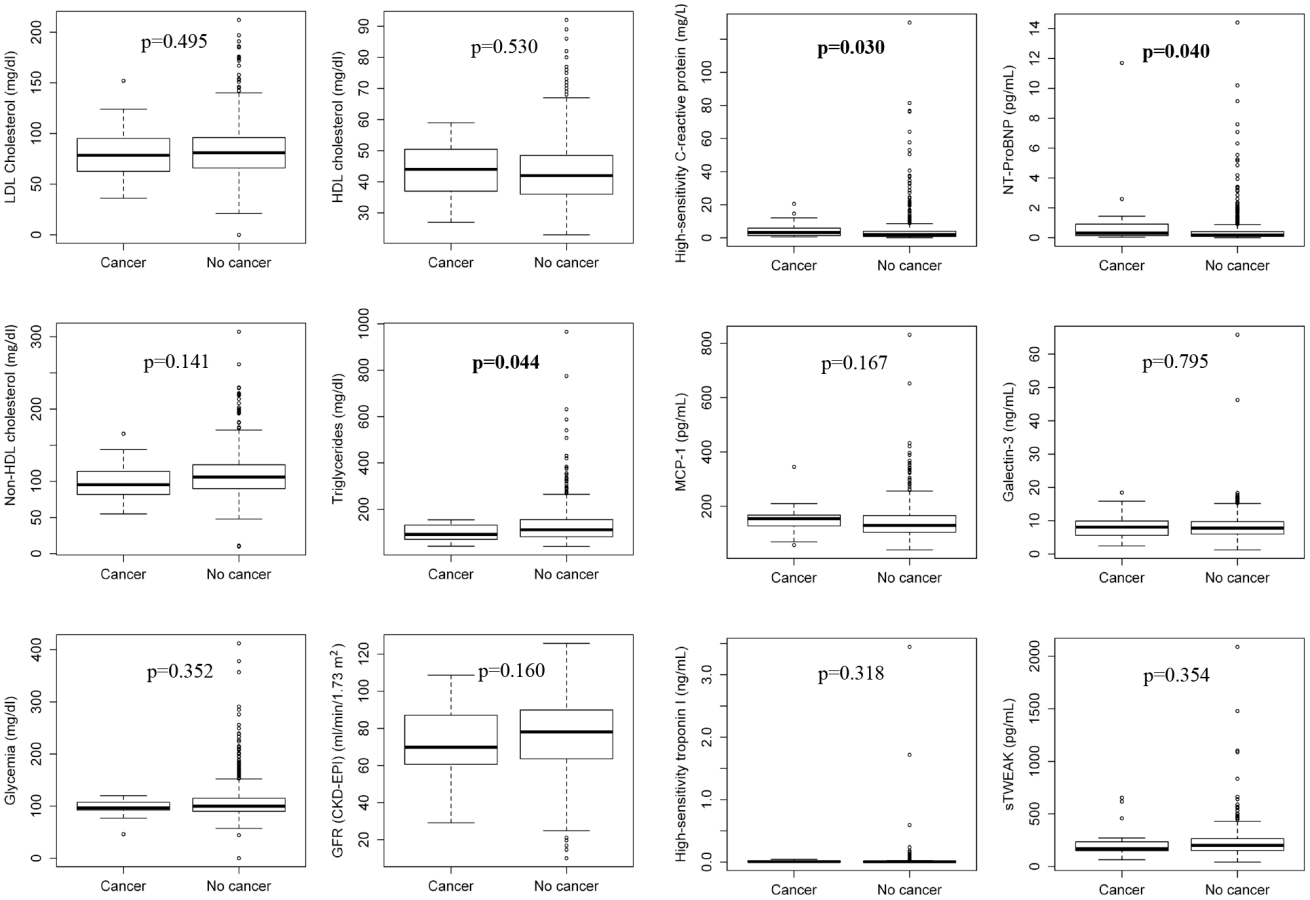
Box graphs showing data of the different analytical variables in patients with and without cancer. Boxes represent the interquartile ranges, with a thick line inside representing the median value. Points outside boxes represent individual values that are not included in the interquartile range. **CKD-EPI:** Chronic Kidney Disease-Epidemiology Collaboration Method; **GFR:** Glomerular Filtration Rate; **HDL:** High-density lipoprotein; **LDL:** Low-density lipoprotein. **NT-proBNP:** N-terminal pro-brain natriuretic peptide; **sTWEAK:** soluble tumor necrosis factor-like weak inducer of apoptosis.

Five patients were lost to follow-up, leaving 699 patients for analysis. Age was 60.0 (52.0, 72.0) years, and there were 75.3% men in the population studied. After 2.15±0.98 years of follow-up, 24 patients developed a new cancer. They were located in lungs (4), prostate (4), larynx (3), urinary bladder (3), colon (2), esophagus (2), breast (2), kidneys (1), and pancreas (1). There was also 1 liposarcoma and 1 amigdalar lymphoma. A histological analysis revealed 12 carcinomas, 9 adenocarcinomas, 1 lymphoma, 1 liposarcoma, and 1 urotelial cancer. Only one lung cancer was a small-cell carcinoma. Patients developing malignancies were older (68.5 [61.5, 75.8] vs 60.0 [52.0, 72.0] years; p = 0.011) and had higher NT-proBNP (302.0 [134.8, 919.8] vs 165.5 [87.4, 407.5] pg/ml; p = 0.040), and high-sensitivity C-reactive protein (3.27 [1.33, 5.94] vs 1.92 [0.83, 4.00] mg/L; p = 0.030), and lower triglyceride (92.5 [70.5, 132.8] vs [112.0 (82.0, 157.0] mg/dl; p = 0.044) plasma levels than those remaining stable, without differences in the other variables studied (Table 1 and Fig 2), including previous or present smoking. The percentage of present smokers at baseline was very low (6.6%).

**Table 1:** Characteristics of patients with and without cancer.

Characteristic	Patients with cancer (N = 24)	Patients without cancer (N = 675)	P Value
**Age (yr)**	68.5 (61.5, 75.8)	60.0 (52.0, 72.0)	**0.011**
**Male sex (%)**	70.8	75.4	0.632
**Body-mass index (Kg/m** ^**2**^ **)**	27.4 (23.8, 30.9)	28.4 (25.8, 30.9)	0.212
**Diabetes (%)**	20.8	24.9	0.812
**Smoker (%)**	83.3	73.5	0.551
**Hypertension (%)**	75.0	65.0	0.386
**Systolic Blood Pressure (mm Hg)**	128.0 (112.5, 140.0)	130.0 (120.0, 140.0)	0.397
**Previous heart failure (%)**	4.2	7.4	1.000
**Peripheral artery disease (%)**	4.2	3.9	0.618
**Cerebrovascular events (%)**	0.0	3.6	1.000
**Ejection fraction < 40% (%)**	20.8	11.6	0.190
**Present or past atrial fibrillation**	8.3	4.9	0.341
**MEDICAL THERAPY**			
**Acetylsalicylic acid (%)**	95.8	91.9	0.713
**Clopidogrel (%)**	66.7	67.9	1.000
**Acenocumarol (%)**	4.2	6.1	1.000
**Statins (%)**	83.3	87.7	0.525
**Oral antidiabetic drugs (%)**	16.7	17.9	1.000
**Insulin (%)**	0.0	6.7	0.393
**Angiotensin-converting enzyme inhibitors (%)**	58.3	55.0	0.836
**Angiotensin receptor blockers (%)**	16.7	16.4	1.000
**Aldosterone receptor blockers (%)**	4.2	5.8	1.000
**Betablockers (%)**	70.8	76.3	0.626
**Diuretics (%)**	25.0	19.3	0.440
**DATA AT LAST ACUTE CORONARY EVENT**			
**STEMI/Non-STEACS (%)**	50.0/50.0	38.4/61.6	0.288
**Number of vessels diseased**	1.0 (1.0, 2.0)	1.0 (1.0, 2.0)	0.911
**Percutaneous interventionism (%)**	83.3	73.2	0.350
**Drug-eluting stent (%)**	33.3	47.6	0.213
**Coronary artery bypass graft (%)**	0.0	5.9	0.390
**Complete revascularization (%)**	58.3	65.3	0.535
**ANALYTICAL DATA**			
**LDL cholesterol (mg/dl)**	78.5 (62.3, 95.0)	81.0 (66.0, 96.0)	0.495
**HDL cholesterol (mg/dl)**	44.0 (37.0, 50.8)	42.0 (36.0, 49.0)	0.530
**Non-HDL cholesterol**	95.5 (82.0, 115.0)	106.0 (90.0, 123.0)	0.141
**Triglycerides (mg/dl)**	92.5 (70.5, 132.8)	112.0 (82.0, 157.0)	**0.044**
**Glycemia (mg/dl)**	96.5 (92.3, 107.8)	100.0 (90.0, 115.0)	0.352
**GFR (CKD-EPI) (ml/min/1.73 m** ^**2**^ **)**	69.8 (58.5, 88.2)	78.1 (63.6, 90.0)	0.160
**High-sensitivity C-reactive protein (mg/l)**	3.27 (1.33, 5.94)	1.92 (0.83, 4.00)	**0.030**
**NT-ProBNP (pg/ml)**	302.0 (134.8, 919.8)	165.5 (87.4, 407.5)	**0.040**
**MCP-1 (pg/ml)**	154.4 (125.1, 167.6)	129.6 (104.2, 165.6)	0.167
**Galectin-3 (ng/ml)**	8.07 (5.59, 10.00)	7.78 (6.02, 9.72)	0.795
**High-sensitivity troponin I (ng/ml)**	0.005 (0.000, 0.018)	0.003 (0.000, 0.010)	0.318
**sTWEAK (pg/ml)**	169.9 (149.0, 237.4)	201.9 (151.9, 265.4)	0.354

**CKD-EPI:** Chronic Kidney Disease Epidemiology Collaboration; **GFR:** glomerular filtration rate; **HDL:** high-density lipoprotein; **LDL:** low-density lipoprotein **MCP-1:** monocyte chemoattractant protein-1; **Non-STEACS:** Non-ST elevation acute coronary syndrome; **NT-Pro-BNP:** Pro-Brain natriuretic peptide; **STEMI:** ST-elevation myocardial infarction; **sTWEAK:** soluble tumor necrosis factor-like weak inducer of apoptosis. Quantitative variables are displayed as median (interquartile range), as they did not follow a normal distribution.

NT-proBNP levels were 301.0 pg/ml in the case of lymphoma, 357.5 (138.8, 803.5) pg/ml in cases of lung cancer, 705.5 (178.0, 1082.0) pg/ml in prostatic cancer, and 291.0 (85.4, 931.0) pg/ml in all the other patients with malignancies, showing no differences among them (p = 0.720 for the comparison between the last 3 groups). NT-proBNP levels were 301.0 (103.8, 919.8) and 303.0 (115.8, 876.5) pg/ml in patients developing carcinoma and adenocarcinoma, respectively (p = 0.808).

A univariate Cox regression analysis revealed that only age (hazard ratio [HR] = 1.036 [95% confidence interval (CI) = 1.002–1.072]; p = 0.039), and NT-proBNP (HR = 1.016 [CI = 1.001–1.033] per 100 pg/ml increment; p = 0.041) and triglyceride (HR = 0.988 [CI = 0.978–0.999]; p = 0.032) plasma levels were associated with a new diagnosis of cancer. All other variables displayed in Table 1 did not reach statistical significance (not shown).

By multivariate Cox regression analysis, only NT-proBNP (HR = 1.030 [CI = 1.008–1.053] per 100 pg/ml increase; p = 0.007) and triglyceride levels (HR = 0.987 [CI = 0.975–0.998]; p = 0.024) remained as independent predictors of a new diagnosis of cancer. There was no difference in the use of statins between both groups (Table 1). No patients in the cancer group were receiving fibrates, as compared to 6.1% in the group that had not developed cancer (p = 0.390).

In four patients, the suspicion of cancer was raised during the first one hundred days of follow-up. These patients showed no significant differences in age, glomerular filtration rate, or NT-proBNP (121.3 [51.8, 700.3] vs 317.0 [175.3, 981.3] pg/ml; p = 0.157), triglyceride (81.5 [61.5, 95.5] vs 92.5 [72.5, 136.7] mg/dl; p = 0.273), glucose, LDL, HDL, non-HDL, high-sensitivity C-reactive protein, MCP-1, galectin-3, high-sensitivity cardiac troponin I, and sTWEAK plasma levels as compared to those presenting later. When Cox multivariate regression analysis was repeated excluding these four patients, NT-proBNP (HR = 1.061 [CI = 1.034–1.088] per 100 pg/ml increase; p<0.001) was the only variable showing independent predictive value for the appearance of new malignancies.

### Relationship of NT-proBNP with other variables

**Fig 3:**
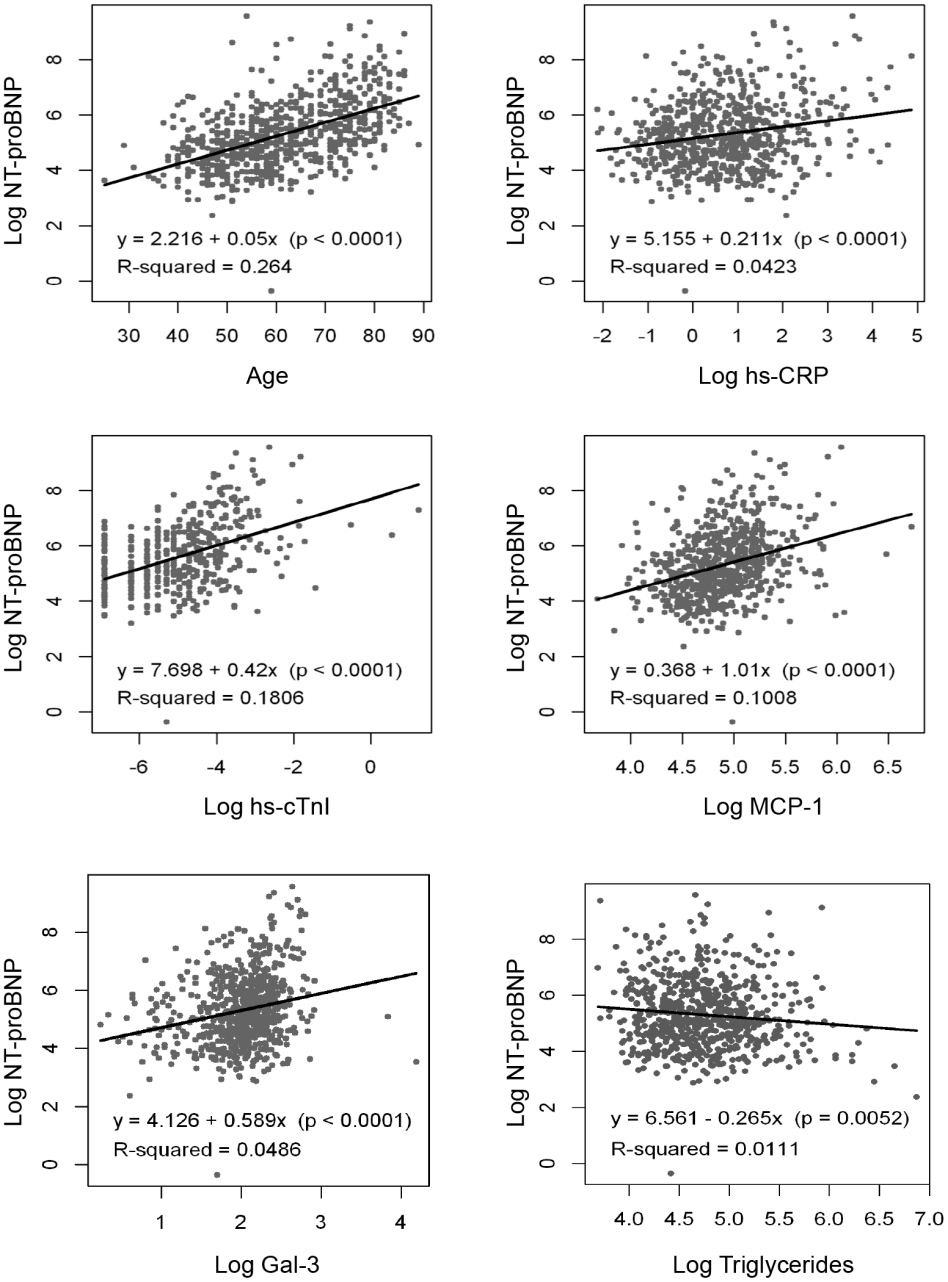
Linear regressions showing the correlations of NT-proBNP with age and other biomarkers studied. Correlations were significant but mild with age, Glomerular Filtration Rate **(GFR)** as assessed by the Chronic Kidney Disease Epidemiology Collaboration Method **(CKD-EPI),** high-sensitivity C-reactive protein **(hs-CRP),** high-sensitivity cardiac troponin I **(hs-cTnI),** monocyte chemoattractant protein-1 **(MCP-1),** galectin-3 **(Gal3),** and triglyceride plasma levels. **Log:** Logarithm.

NT-proBNP levels were higher in women than in men (239.0 [130.5, 561.5] vs 156.0 [82.6, 359.0] pg/ml; p<0.001), and in patients with hypertension (225.0 [114.0, 530.0] vs 111.5 [62.7, 223.0] pg/ml; p<0.001) and with previous atrial fibrillation (886.0 [316.0, 1860.0] vs 162.0 [85.9, 378.0] pg/ml; p<0.001) as compared with those not suffering these conditions. Linear regression analyses were performed to assess the extent to which NT-proBNP levels could be explained by other variables. Given that the distribution of all biomarkers was asymmetric, we used logarithms for this analysis. NT-proBNP showed significant but mild correlations with age, glomerular filtration rate, and with plasma levels of high-sensitivity C-reactive protein, high-sensitivity cardiac troponin I, MCP-1, galectin-3, and triglycerides, (Fig 3), indicating that these variables did not explain most of NT-proBNP variability. The correlation with body-mass index and sTWEAK was not significant (R^2^ = 0.002, p = 0.305; and R^2^ = 0.000, p = 0.856, respectively). By multivariate linear regression analysis all the variables exhibiting a significant relationship with NT-proBNP at univariate analysis were significant, with the exception of galectin-3 (Table 2). These variables explained about 41% of the variations of NT-proBNP.

**Table 2:** Multivariate linear regression analysis showing the influence of other variables on N-terminal probrain natriuretic peptide levels.

	Coefficient	95% CI	P value
**Intercept**	**5.730**	3.839, 7.621	<0.001
**Age**	**0.022**	0.013, 0.031	<0.001
**Log hs-CRP**	**0.139**	0.060, 0.218	<0.001
**Log hs-cTnl**	**0.307**	0.233, 0.381	<0.001
**Log MCP-1**	**0.358**	0.090, 0.626	0.009
**Log Gal-3**	**0.128**	-0.098, 0.354	0.266
**Log TG**	**-0.264**	-0.458, -0.070	0.008
**GFR (CKD-EPI)**	**-0.012**	-0.018, -0-006	<0.001

R^2^: 0.407; **CI:** Confidence Interval; **CKD-EPI:** Chronic Kidney Disease Epidemiology Collaboration; **Gal-3:** Galectin-3; **GFR:** glomerular filtration rate; **Hs-CRP:** high sensitivity C-reactive protein; **hs-cTnI:** high sensitivity-core troponin I; **Log:** Logarithm; **MCP-1:** monocyte chemoattractant protein-1; **TG:** Triglycerides.

### NT-proBNP in the prediction of cancer, heart failure and death

In addition to 24 cancers, 19 patients developed heart failure, and 23 died, yielding a total of 66 events. However, some patients had two events: 9 presented heart failure and death and 6 had developed a new malignancy and death. So the total number of patients having at least one event was 51.

The cause of death was heart failure in 5 cases, cancer in 4, unknown in 5, and sudden death in 3. Pancreatitis, infection, renal failure, digestive bleeding, bowel ischemia, and cholecystitis accounted for 1 death each.

At multivariate Cox regression analysis, NT-proBNP was an independent predictor of the outcome of new cancer diagnosis, heart failure or death, along with age and use of acenocumarol and insulin (Table 3).

**Table 3:** Multivariate Cox regression analysis for the incidence of cancer, heart failure, or death.

	**Hazard Ratio**	**95% CI**	**P**
**Age**	**1.051**	1.020, 1.084	0.001
**Acenocumarol**	**2.415**	1.112, 5.243	0.026
**Insulin**	**3.085**	1.314, 7.241	0.010
**NT-proBNP**	**1.038** *	1.023, 1.052*	<0.001

**CI:** confidence interval. **NT-proBNP:** N-terminal pro-brain natriuretic peptide.

*Increase in risk per increment of 100 pg/mL plasma concentration.

## Discussion

Patients with CAD have increased probabilities of developing malignancies. This is so because the incidence of cancer increases with age [1], tobacco consumption and some dietary patterns that promote CAD [2,3]. Thus, predicting the risk of cancer in patients with CAD could be of interest.

NT-proBNP is derived from pre-proBNP secreted by the ventricular myocardium [14] and is used mainly for the diagnosis of heart failure [15]. In addition, it may predict the development of heart failure and death in patients with cardiovascular disease [13,14]. However, NT-proBNP could have predictive power beyond cardiovascular risk. In fact, this biomarker is associated with total death in patients with cardiovascular diseases [14] and in elderly subjects [16–19].

We describe for the first time that NT-proBNP is also an independent predictor of the appearance of malignancies in patients with CAD. Until now, natriuretic peptides have been investigated as predictors of cardiac toxicity secondary to chemotherapy [20]. More recently, a possible relationship between natriuretic peptide plasma levels and cancer itself has been suggested. In fact, patients with cancer may have elevated BNP levels in the absence of volume overload [6,21]. In patients over 40 years of age with previous elective non-cardiac surgery, NT-proBNP levels equal to or higher than 125 pg/ml were independently associated with a diagnosis of lung cancer after excluding patients with heart failure, CAD, and other conditions known to affect this biomarker [22]. Although natriuretic peptides had been shown to be secreted by small-cell lung cancer [4,5], only 15% of the cases reported in this study, and one in the present paper, had this type of cancer, suggesting that other tumors could also produce NT-proBNP. In addition, NT-proBNP was an independent predictor of survival in patients with non-Hodgkin lymphoma treated with chemotherapy, after excluding those who were candidates for palliative chemotherapy and those with human immunodeficiency virus infection [23]. Twelve percent had previous heart disease and the median ejection fraction was 65%. Of interest, NT-proBNP levels were associated with the involvement of two or more extranodal sites, suggesting a potential relationship with the stage of this malignancy. Similarly, increased NT-proBNP plasma levels predict the progression of metastatic renal carcinoma [24]. Although hematological and lung cancer seem to have been especially related to NT-proBNP levels, in our series there were no differences in plasma levels of this biomarker among these groups of patients with cancer, and the groups of patients with all other tumors. Nevertheless, in our series there was only one case of lymphoma. Also, we found no differences among the various histopathological types of cancer, although the small number of patients analyzed in each subgroup limits the validity of this observation.

At present, it is well known that natriuretic peptides may be produced by cancer cells. In this regard, small-cell lung cancer may secrete both pro-atrial natriuretic peptide and BNP [4,5]. Also, BNP is expressed both in normal adrenal glands and in adrenal tumors [25], suggesting that natriuretic peptides may have other roles unrelated to the cardiovascular system. ProBNP synthesis may be stimulated by several proinflammatory cytokines, including tumor necrosis factor-α and some interleukins [26]. Tumor necrosis factor-α and interleukin-6 are expressed in Reed-Stemberg cells from patients with Hodgkin lymphoma [27,28]. Moreover, they may predict outcome in diffuse large B-cell lymphomas [29–31] and, as explained, are increased in malignancies at advanced stages [23]. However, the specific cause of the elevation of natriuretic peptide plasma levels seen in cancer has not been elucidated. In recent years, it has been demonstrated that natriuretic peptides, or compounds with similar activity, decrease the number of several cancer cells in vitro through a reduction of DNA synthesis [32] and inhibition of c-Fos and c-Jun protooncogenes [33], inhibit lung metastases and skin carcinogenesis in animal models [34,35], and diminish the expression of vascular endothelial growth factor and that of its receptor VEGFR2, thus having the potential to control vasculogenesis [36]. One work has shown opposite effects of natriuretic peptides on carcinogenesis depending on their concentrations [37]. In that paper, atrial natriuretic peptide enhanced proliferation of human gastric cells in vitro at low concentrations, but inhibited their proliferation through cyclic guanosine 3’5’ monophosphate-dependent or -independent pathways when it was present at high concentrations [37]. Then, given that most data suggest an anti-cancer effect of natriuretic peptides, the possibility exists that their production by cancer cells represents a negative feed-back mechanism to control tumor growth. In this case, the NT-proBNP elevation shown here would only be a response to the existence of malignancies. Nevertheless, the fact that natriuretic peptides are related with mechanisms of cancer which are common to multiple malignancies would be in agreement with our findings, since we did not observe significant differences in NT-proBNP levels among patients with different types of cancer.

In our population, low triglyceride levels were also independently associated with the development of malignancies in the absence of significant differences in lipid-lowering therapy. Although there is no evidence of a causal relationship [38], low cholesterol levels are associated with cancer even at preclinical stages [39]. This fact could raise the suspicion that some cancers were present at the moment of blood extraction. This is possible, given that there is a lapse of time between the moment when cancer develops and the first signs or symptoms of the disease appear. We therefore hypothesize that increased NT-proBNP levels could indicate the existence of a tumor that is not made evident using the tools available in current clinical practice. In that case, NT-proBNP could be useful for early detection of malignancies. Nevertheless, all patients showing signs or symptoms of cancer at baseline were excluded even if the diagnosis was confirmed later. Moreover, we performed a second multivariate analysis excluding patients in whom the first symptoms or signs of cancer presented during the first one hundred days of follow-up. In this way we tried to separate the cases of patients that could theoretically have noticed some abnormalities in the weeks before blood extraction for the study though did not report them to their doctors, perhaps because the patients considered them unimportant. In this analysis, NT-proBNP levels were the only independent predictor of future cancer diagnosis, thereby confirming that this biomarker may predict a future diagnosis of cancer in cases that are asymptomatic at the moment of blood extraction. Further work is needed to elucidate if it predicts the development of new cancer or simply detects subclinical tumors.

The possibility of confounding results because NT-proBNP was acting as a marker of heart failure is highly improbable. First, all patients where stable and had not heart failure when blood extraction was performed. Second, the presence of previous heart failure was very low and not different between both groups. Finally, none of the patients who developed cancer in our series suffered heart failure during follow-up. In addition, the possibility of any other relationship of NT-proBNP levels with the underlying CAD is also improbable, as in the multivariate analysis we have controlled for a complete set of variables related with this condition.

Some factors may influence NT-proBNP levels. Increasing age, female sex, hypertension, atrial fibrillation and decreased glomerular filtration rate are associated with high plasma levels of this biomarker, while increasing body-mass index may be related to lower NT-proBNP [14, 40, 41]. In this study, NT-proBNP levels were confirmed to be higher in women than in men, and to increase with age and impaired renal function. However, NT-proBNP remained as an independent predictor of the development of cancer after controlling for all these variables. Of interest, increasing age is associated with high NT-proBNP levels and cancer. Although the increase in natriuretic peptide levels could favor the development of cancer, most of the evidence that we have found in the literature suggests that they seem to have anti-cancer effects [32–37] and therefore could work as a negative feed-back when malignancies develop.

Finally, NT-proBNP plasma levels were able to predict the development of the composite outcome integrating cancer, heart failure and death. This is a relevant feature, since patients are concerned about any event that could shorten their lives or limit their quality of life regardless of whether they are related to any one medical subspecialty or several of them. If these results are confirmed in future studies, we will be able to enter a new era in the investigation of biomarkers focusing on the prediction of disorders that can share pathogenic aspects and have a serious impact on patient lives, even although they are not related to a single medical specialty.

This work has some limitations. 1) The number of patients diagnosed with cancer during follow-up was small, and this finding should be confirmed in larger populations. Similarly, it seems unwise to attempt to establish a cut-off point of NT-proBNP levels for the diagnosis of future cancer given the limited number of cancer cases reported in this paper. 2) The lack of association between tobacco and cancer diagnosis could be surprising. However, given the previous diagnosis of CAD, most patients had given up smoking, thus reducing the possibility of finding such a relationship in this population. 3) Finally, plasma withdrawn during the acute index event has not been studied, because at this point the population was composed of patients with ST-elevation myocardial infarction and Non-ST-elevation Acute Coronary Syndrome. These are clinical entities with differences in the pathophysiology and management. Given this heterogeneity, mixing plasma findings at this stage could have introduced a bias in the results.

## Conclusions

NT-proBNP is an independent predictor of malignancies in patients with stable CAD. If these findings are confirmed in further studies, this biomarker could be helpful in assessing the global prognosis in patients with CAD.

## Supporting Information

S1 Dataset Database. (XLS)
